# Effects of beetroot juice supplementation on vascular functional and structural changes in aged mice

**DOI:** 10.14814/phy2.15755

**Published:** 2023-06-20

**Authors:** Masashi Tawa, Keisuke Nakagawa, Mamoru Ohkita

**Affiliations:** ^1^ Department of Pathological and Molecular Pharmacology, Faculty of Pharmacy Osaka Medical and Pharmaceutical University Osaka Japan

**Keywords:** aging, beetroot, endothelial dysfunction, medial thickening, nitrate, oxidative stress

## Abstract

This study investigated whether beetroot juice (BRJ) ingestion ameliorates aging‐induced functional and structural changes in vasculature. Aged mice (98–100 weeks old) were supplemented with BRJ (nitrate: 3.5 mmol/L) or drinking water for 4 weeks and compared with young mice (12–15 weeks old). The vasorelaxant response of isolated aortas to acetylcholine was markedly weaker in aged mice than in young mice, but the attenuated relaxation was significantly improved in BRJ‐supplemented aged mice. The acetylcholine‐induced relaxation was completely abolished by *N* ^ω^‐nitro‐l‐arginine methyl ester in all groups. Additionally, the response to sodium nitroprusside was comparable among the three groups. The aortic medial thickness was significantly greater in aged mice than in young mice, and BRJ supplementation did not suppress this thickening. Plasma nitrate levels were significantly higher in BRJ‐supplemented aged mice than in non‐supplemented aged mice. Conversely, non‐supplemented aged mice had high plasma levels of thiobarbituric acid‐reactive substances, but the levels were suppressed in BRJ‐supplemented aged mice. These findings suggest that BRJ ingestion improves vascular endothelial dysfunction associated with aging, at least in part, by enhancing nitric oxide bioavailability and reducing oxidative stress. Therefore, beetroot ingestion may be a highly useful self‐medication option to prevent vascular aging.

## 1 INTRODUCTION

The function and structure of blood vessels change with age; this is referred to as vascular aging (Thijssen et al., 2016). The molecular mechanisms of vascular aging have been extensively validated, and endothelial dysfunction has been identified as a characteristic feature (Camici et al., 2015; Ungvari et al., 2018). Nitric oxide (NO), synthesized by endothelial NO synthase (eNOS), mediates several biological functions, including vasodilation, antithrombosis, anti‐fibrosis, and anti‐proliferation (Moncada et al., 1991; Vanhoutte et al., 2016). Therefore, a decreased NO bioavailability is considered the pathogenesis of endothelial dysfunction, especially in large blood vessels (Cyr et al., 2020; Münzel et al., 2008). Importantly, elderly individuals with poor flow‐mediated dilation (FMD), an index of NO‐mediated endothelial function, are at an increased risk of cardiovascular disease (Kabutoya et al., 2018; Yeboah et al., 2007). Additionally, there is a strong correlation between increased carotid artery intima‐media thickness and the incidence of cardiovascular events in the elderly (Nagai et al., 2013; O'Leary et al., 1999). These findings imply that the prevention of vascular aging is important for longevity in older adults.

Dietary nitrate can increase NO bioavailability through the nitrate–nitrite–NO pathway. Nitrate is converted into nitrite by oral bacteria and then into NO by certain proteins and enzymes, such as deoxyhemoglobin and xanthine oxidoreductase (Kapil et al., 2020). Therefore, nitrate intake is likely to confer cardiovascular benefits. Supplementation with sodium nitrate for several weeks ameliorated vascular endothelial dysfunction in aged mice (Rammos et al., 2015) and aged rats (Hezel et al., 2016). Similar findings have been obtained in humans, where elderly individuals who ingested sodium nitrate for 4 weeks reported better FMD than those who ingested sodium chloride (Rammos, Hendgen‐Cotta, Sobierajski, et al., 2014). Thus, dietary nitrate may represent a promising therapy for the treatment of vascular aging in old age.

Beetroot (*Beta vulgaris L*.) is a nitrate‐rich vegetable (Chhikara et al., 2019) and therefore elicits similar effects as nitrate when ingested (Bhaswant et al., 2017; Morris Jr et al., 2019; Velmurugan et al., 2013). Several studies in older adults without cardiovascular disease provided evidence that beetroot juice (BRJ) consumption for 4 weeks improves vascular function in conduit arteries in a nitrate‐dependent manner (Casey & Bock, 2021; Jones et al., 2019). However, only one study has reported the effect of BRJ consumption on age‐related vascular endothelial dysfunction (Jones et al., 2019), and there are no reports on age‐related vascular remodeling. Therefore, further evidence is needed to conclude whether beetroot is useful for the suppression of vascular aging in senescence. In this study, we investigated the effects of BRJ supplementation on vascular endothelial function and morphology in aged mice.

## 2 MATERIALS AND METHODS

### 2.1 Animals

All animal experiments were conducted according to the Guide for the Care and Use of Laboratory Animals (8th edition, 2011). Eight male C57BL/6J mice (8 weeks old) and 20 male B6J‐Aged mice (95 weeks old) were purchased from Charles River Laboratories Japan, Inc. and housed at the Animal Testing Facility, Faculty of Pharmacy, Osaka Medical and Pharmaceutical University until experimentation. Young and aged animals were used in the experiments at 8–11 and 98–100 weeks of age, respectively. The mice were allowed access to food and water ad libitum and housed four to five per cage in a light‐controlled room with a 12‐h light–dark cycle. The Experimental Animal Research Committee of Osaka University of Pharmaceutical Sciences provided ethical approval for the use of laboratory animals in this study (Permit No. 59/2021, approved March 31, 2021); Osaka University of Pharmaceutical Sciences in use until March 31, 2021 is the former name of Osaka Medical and Pharmaceutical University.

### 2.2 Experimental protocols

Three experimental groups were studied: (1) young group—young mice were supplemented with water; (2) aged group—aged mice were supplemented with water; and (3) BRJ group—aged mice were supplemented with BRJ (containing 3.5 mmol/L nitrate with a dilution factor of 20; Beet It Organic Shot, James White Drinks Ltd.). Only a single lot of BRJ was used in this study. The solutions were changed every 1–2 days, and supplementation was maintained for 4 weeks. The dilution factor was determined based on the nitrate concentrations used in previous studies (Carlström et al., 2010; Hezel et al., 2016; Rammos et al., 2015). The average nitrate intake per day in the BRJ group was 521 μmol/kg/day; this was calculated assuming that each mouse in a cage received an equal amount of BRJ. Body weight was measured once per week to calculate the daily nitrate intake for each week (Table S1) and the values, thus, obtained were averaged per 4 week and then per group.

After 4 weeks, the mice were deeply anesthetized with intraperitoneal sodium pentobarbital (50 mg/kg; Kyoritsu Seiyaku Co.), and blood samples were collected from the inferior vena cava. The mice were then euthanized, and the thoracic aortas were excised and dissected into 3‐ to 5‐mm rings for organ bath studies. The redundant portions were subjected to histological analyses.

### 2.3 Organ bath experiments

The aortic rings were fixed using stainless steel wires in an organ bath filled with Krebs‐Ringer bicarbonate solution (37°C, pH 7.4) and continuously bubbled with 95% O_2_ and 5% CO_2_. The upper wire was connected to the lever of a force‐displacement transducer (TB‐612 T, Nihon Kohden), and the lower wire was fixed at the bottom of the organ bath. Isometric contractions and relaxations were recorded using the PowerLab Data Acquisition System (PL3508/P, ADInstruments). The resting tension was adjusted to 1.0 g, and all rings were allowed to equilibrate in the bathing medium for 60–90 min, during which the solution was replaced every 15 min.

After equilibration, phenylephrine (1 μM; Tokyo Chemical Industry Co., Ltd.) was added to the organ bath to induce contractions. When the contractions reached a stable plateau, acetylcholine (ACh, 100 pM–10 μM; FUJIFILM WAKO Pure Chemical Co.), A23187 (3 nM–1 μM; Cayman Chemical Co.), and sodium nitroprusside (SNP, 100 pM–10 μM; Sigma‐ Aldrich Co. LLC) were administered cumulatively into the organ bath. At the end of each experiment, papaverine (100 μM; Dainippon‐Sumitomo Pharma Co.) was added to induce maximal relaxation, which was taken as 100% relative to the relaxations induced by agonists. In some cases, the same procedure was conducted in the presence of *N* ^ω^‐nitro‐l‐arginine methyl ester (l‐NAME, 100 μM; Tokyo Chemical Industry Co., Ltd.), a NO synthase inhibitor.

### 2.4 Histological analysis

The aortas were fixed in 10% phosphate‐buffered formalin and embedded in paraffin. Tissue sections with a thickness of 4 μm were obtained and stained using the Elastica van Gieson method. Morphometric analysis of each segment was performed using an image analyzer (cellSens, Olympus Co., Ltd.). The borders of the lumen, internal elastic lamina, and external elastic lamina were traced, and their circumferences were measured. The medial wall thickness percentage was calculated and expressed as follows: medial thickness (%) = [(external diameter minus internal diameter)/external diameter] × 100.

### 2.5 Biochemical marker analysis

Heparinized blood samples were centrifuged at 3000 rpm for 10 min at 4°C, and the plasma obtained was stored at −80°C until use. The plasma was mixed with methanol (1:1, v/v) and centrifuged at 10,000 rpm for 10 min at 4°C. The resulting supernatant was used for the following experiments.

For the analysis of nitrite and nitrate, the supernatant was diluted with the mobile phase (NOCARA, Eicom) and injected into an ENO‐20 NOx Analyzer (Eicom). Nitrite and nitrate levels were calculated by comparison with the results obtained from standard solutions (NO‐STD, Eicom).

In the analysis of the thiobarbituric acid‐reactive substances (TBARS), the supernatant was incubated for 45 min at 65°C with thiobarbituric acid (TBA) under acidic conditions. The malondialdehyde (MDA)‐TBA adduct was measured colorimetrically at 532 nm. The TBARS levels were calculated by interpolation of a standard curve of MDA. An assay kit (FR40, Oxford Biomedical Research, Inc.) was used for these measurements.

### 2.6 Statistical analysis

All values are presented as the mean ± SEM. Concentration–response curves were fitted by nonlinear regression analysis using the GraphPad Prism software (version 7.0, Graph Pad Software Inc.). The concentration–response curves were analyzed using two‐way repeated‐measures analyses of variance (ANOVA) and Bonferroni post‐hoc tests. The medial thickness, lumen diameter, and levels of nitrite, nitrate, and TBARS were compared using the Bonferroni multiple comparisons test after one‐way ANOVA. A value of *p* < 0.05 was considered significant.

## 3 RESULTS

### 3.1 Effects of BRJ supplementation on vascular reactivity

**FIGURE 1:**
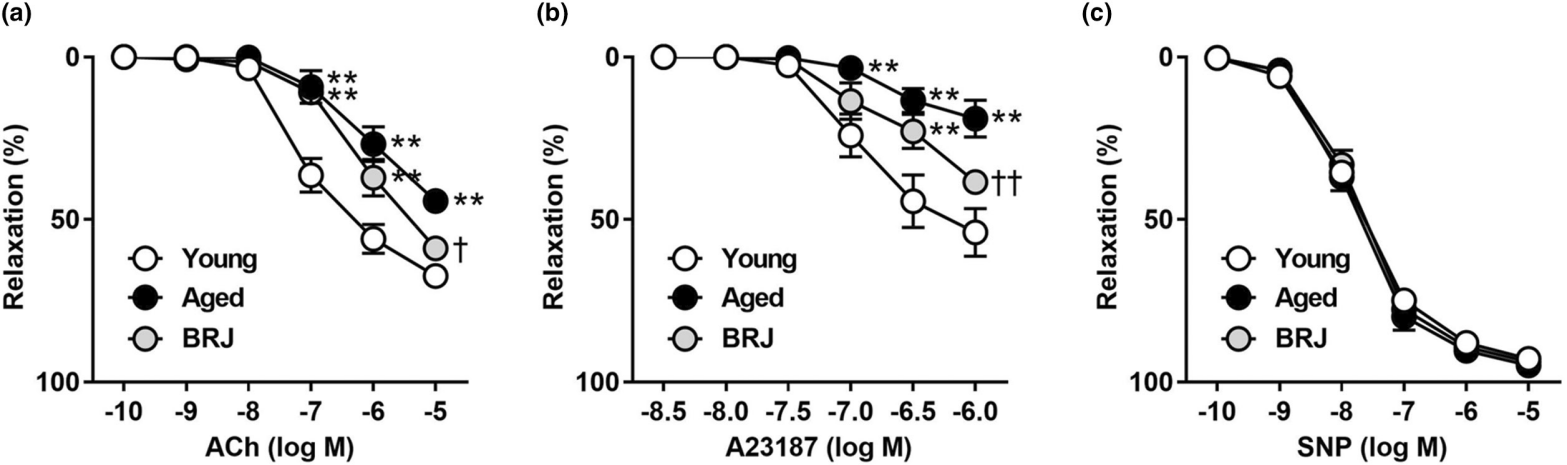
Concentration–response curves to ACh (a), A23187 (b), and SNP (c) in young (white circle), aged (black circle), and BRJ‐supplemented aged mouse aortas (gray circle). Each point and bar represent the mean ± SEM values of 8–10 experiments. **p < 0.01, compared to the young group; ^†^ *p* < 0.05 and ^††^ *p* < 0.01, compared to the aged group. ACh, acetylcholine; BRJ, beetroot juice; SNP, sodium nitroprusside.

The addition of ACh (endothelium‐dependent vasodilator) produced a concentration‐dependent relaxation. The ACh‐induced relaxation was significantly reduced in the aged group compared with that in the young group, and the attenuation was less in the BRJ group (Figure 1a). The relaxant response to ACh was abolished by treatment with l‐NAME in all groups (data not shown). As is the case with the response to ACh, the aged group showed an impaired A23187 (endothelium‐dependent but receptor‐independent vasodilator)‐induced relaxation, which was partly reversed by BRJ supplementation (Figure 1b). The concentration‐related relaxation produced by SNP (endothelium‐independent vasodilator) was comparable among the three groups (Figure 1c).

### 3.2 Effects of BRJ supplementation on vascular morphology

**FIGURE 2:**
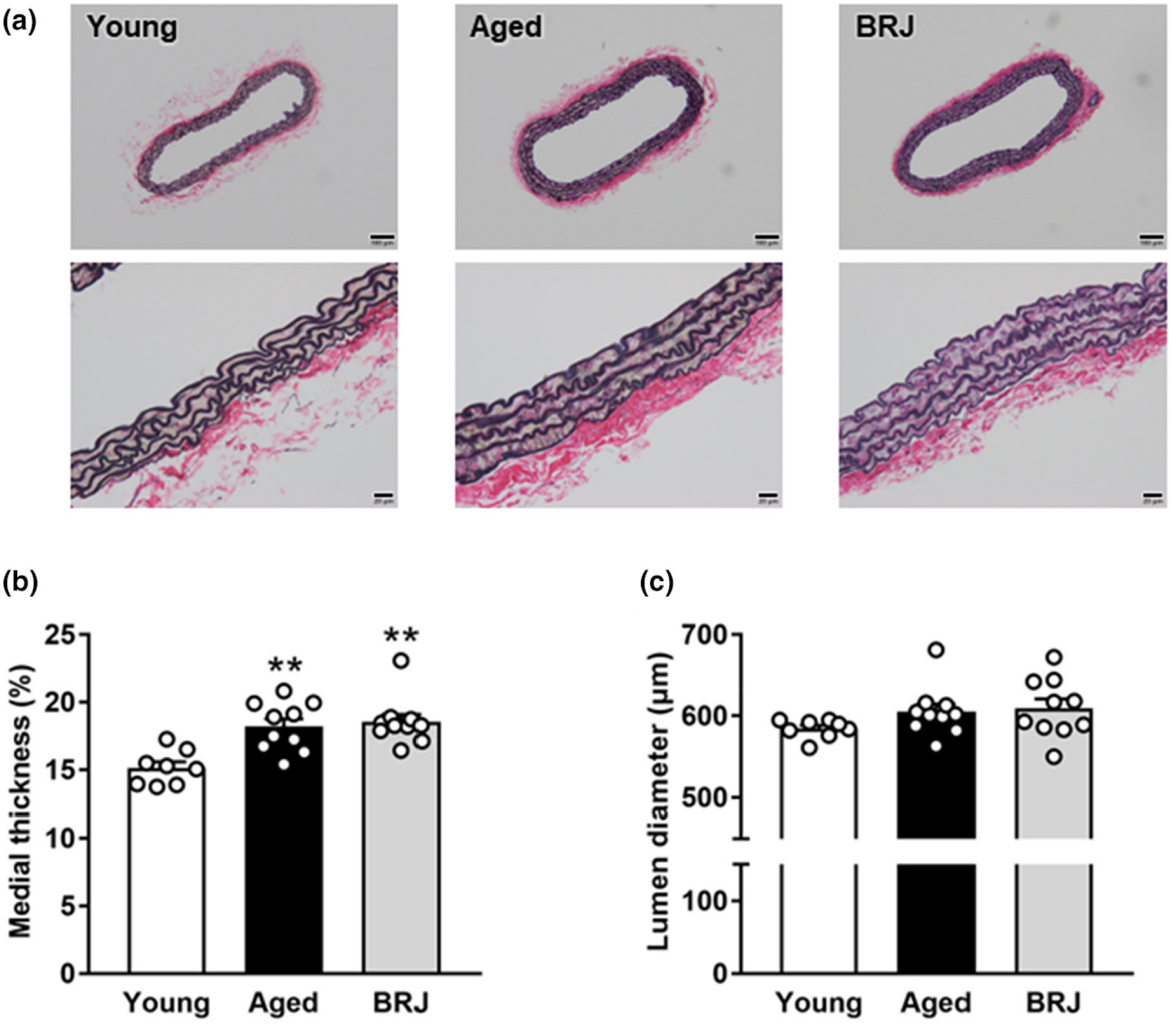
Typical images (a), medial thickness (b), and lumen diameter (c) in young (white column), aged (black column), and BRJ‐supplemented aged mouse aortas (gray column). Scale bars, 100 μm (upper panels) and 20 μm (bottom panels). Each point represents an individual value; each column and bar represent the mean ± SEM values of 8–10 experiments. **p < 0.01, compared to the young group. BRJ, beetroot juice.

Apparent medial thickening was detected in the aged group compared with that in the young group. The degree of medial thickening was similar between the aged and BRJ groups (Figure 2a,b). Lumen diameters were similar across all groups (Figure 2c).

### 3.3 Effects of BRJ supplementation on nitrite and nitrate levels

**FIGURE 3:**
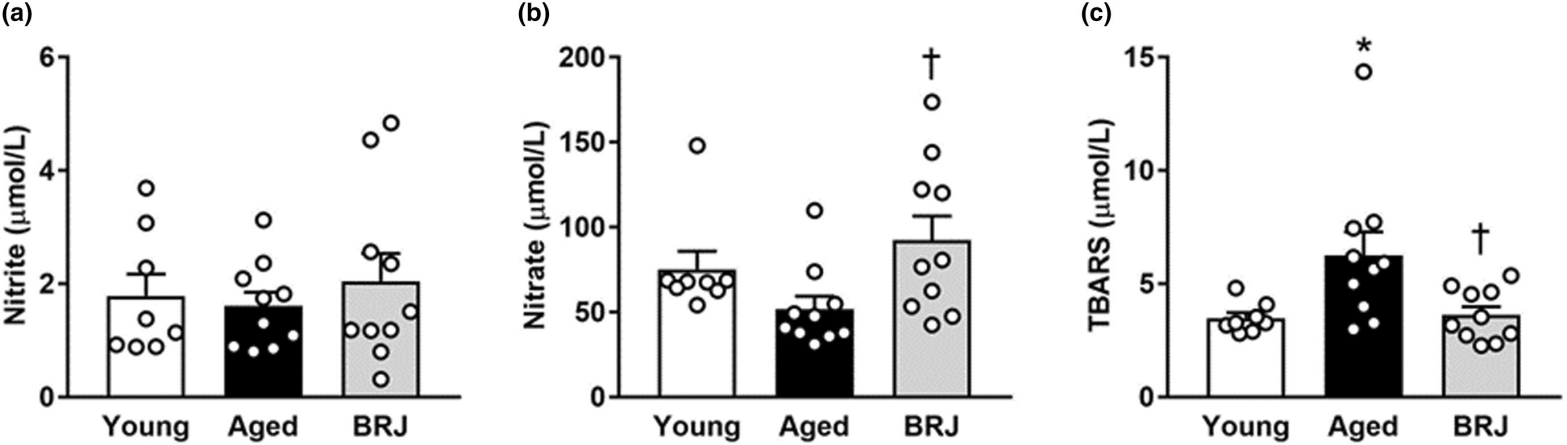
Plasma nitrite (a), nitrate (b), and TBARS levels (c) in young (white column), aged (black column), and BRJ‐supplemented aged mice (gray column). Each point represents an individual value; each column and bar represent the mean ± SEM values of 8–10 experiments. *p < 0.05, compared to the young group; ^†^ *p* < 0.05, compared to the aged group. BRJ, beetroot juice; TBARS, thiobarbituric acid‐reactive substances.

Plasma nitrite and nitrate levels are indicators of NO bioavailability. There was no significant difference in plasma nitrite levels among the three groups (Figure 3a). The nitrate levels in aged mice tended to be lower than those in young mice; however, this difference was not statistically significant. The levels were significantly higher in the BRJ group than in the aged group (Figure 3b).

### 3.4 Effects of BRJ supplementation on TBARS levels

Plasma TBARS levels are markers of systemic oxidative stress, which is defined as the excess production of reactive oxygen species (ROS). A significant increase in the TBARS levels was observed in aged mice compared with that in young mice but not in BRJ‐supplemented mice (Figure 3c).

## 4 DISCUSSION

This study is the first to comprehensively evaluate the effects of beetroot on age‐related vascular changes from both the aspects of functionality and morphology. Beetroot was found to be beneficial for age‐related vascular endothelial dysfunction but not for vascular wall thickening.

The functional integrity of the vascular endothelium was assessed using two endothelium‐dependent agonists. ACh binds to its receptors on the endothelium to increase the intracellular Ca^2+^ concentration, whereas A23187 directly elevates this concentration, both of which activate eNOS to produce and release NO (Furchgott, 2020; Vanhoutte et al., 2016). ACh‐ and A23187‐induced relaxation were attenuated in aortas obtained from aged mice, suggesting endothelial dysfunction with causes downstream of the receptor. In addition, as l‐NAME abolished the relaxant response to ACh in both young and aged mice, this impairment was thought to be due to the decreased NO bioavailability. Furthermore, aged mice had a normal response to SNP, a NO‐donating drug. Thus, the dysfunction observed possibly occurred at the endothelial level and not at the smooth muscle. Similar findings that aging induces vascular endothelial dysfunction with reduced NO bioavailability have been reported in many studies (de Sotomayor et al., 2005; Soltis, 1987; Takenouchi et al., 2009; Tschudi et al., 1996). Notably, BRJ supplementation in aged mice restored the relaxant responses to ACh and A23187 without enhancing relaxation in the presence of l‐NAME. This indicates that BRJ supplementation improved NO‐dependent endothelial function and corresponds with a previous report showing that daily BRJ consumption is associated with enhanced FMD in healthy, older adults (Jones et al., 2019). As an aside, evidence that beetroot is beneficial for endothelial function has been obtained for various vascular diseases, including hypertension (Asgary et al., 2016; Kapil et al., 2015) and hypercholesterolemia (Velmurugan et al., 2016). Overall, beetroot could be a promising option for preventing age‐related vascular endothelial dysfunction.

Vascular wall thickening is a well‐known hallmark of age‐related vascular remodeling (Adrian et al., 2008; Coquand‐Gandit et al., 2017; Wheeler et al., 2015). The aged mice used in this study also showed pronounced medial thickening without affecting the lumen diameter, implying the occurrence of outward hypertrophic remodeling. As BRJ supplementation has beneficial effects on vascular wall thickening (Morselli et al., 2021; Tawa et al., 2021, 2022), we expected that age‐related vascular remodeling would also be suppressed in this study. However, BRJ supplementation did not reverse the medial thickening observed in aged mice. In this regard, similar findings that intervention improves vascular endothelial dysfunction but not medial thickening have been reported in other study (Bulckaen et al., 2008). Therefore, it is unsurprising that the effects on endothelial dysfunction and vascular remodeling are not parallel. The aged mice used in this study are already in the completion stage of aortic wall thickening (De Moudt et al., 2022), and BRJ supplementation may have the effect of suppressing the development of thickening but not the effect of regressing it. Alternatively, it may have been necessary to continue the supplementation for a longer period for the regression effect to appear. This is a topic for future research.

Some evidence suggests that BRJ supplementation increases NO levels in mice (Ferguson et al., 2020; Tropea et al., 2020). In this study, BRJ supplementation enhanced plasma nitrate levels in aged mice. As xanthine oxidoreductase, a key enzyme responsible for NO production from nitrate, is upregulated with aging (Battelli et al., 2020), it is reasonable to deduce that BRJ supplementation was associated with increased NO levels in this study. NO can inhibit mitochondrial ROS production via S‐nitrosation of the mitochondrial respiratory chain complex I enzyme (Ceaser et al., 2003; Shiva et al., 2007). ROS oxidizes tetrahydrobiopterin, an essential cofactor required for eNOS activity, leading to eNOS uncoupling (Förstermann & Li, 2011). Certain ROS can also react directly with NO generated in the endothelium to eliminate its bioactivity (Ritchie et al., 2017). Thus, inhibition of ROS production results in increased NO bioavailability. In addition, Rammos et al. (2015) used microarray analyses to show that nitrate supplementation alters the gene expression and transcriptional profiles of pathways related to the antioxidant pathway in aortas of aged mice. Therefore, we hypothesized that BRJ supplementation might have suppressed ROS production. As expected, the results showed that BRJ supplementation normalized increased TBARS levels associated with aging. Overall, these results suggested that BRJ supplementation in aged mice suppresses oxidative stress and increases NO bioavailability; this would be the mechanism by which endothelial function was improved. As supporting evidence for this view, several studies have reported a negative correlation between plasma TBARS levels and NO bioavailability in healthy older adults (Di Massimo et al., 2006) and a positive correlation between aging‐associated oxidative stress and a decline in endothelium‐dependent vasodilation (Donato et al., 2007).

We focused on endothelial dysfunction and medial thickening in this study, but vascular stiffness is another important factor affecting vascular health. In this regard, acute BRJ supplementation has been reported to reduce vascular stiffness as assessed by the augmentation index (AIx75) in healthy, older adults (Walker et al., 2019). However, the most important issue is the kind of effect brought about by long‐term ingestion. Unfortunately, to our knowledge, no data are available regarding the chronic effects of BRJ supplementation on age‐related increases in vascular stiffness. Incidentally, chronic dietary nitrate supplementation improves vascular stiffness in older adults (Rammos, Hendgen‐Cotta, Pohl, et al., 2014; Rammos, Hendgen‐Cotta, Sobierajski, et al., 2014). Therefore, beetroot may have long‐term beneficial effects. Ongoing (clinicalTrials.gov identifier: NCT03617302) and future basic and clinical studies may provide answers to this unresolved issue.

One limitation of this study is that it was conducted using a single BRJ variant, which contained 2% lemon juice. Interestingly, combined supplementation with vitamin C has been reported to enhance the effect of BRJ (Ashor et al., 2020). Therefore, the effects of BRJ with and without lemon juice may be slightly different. Moreover, the composition and content of nutrients in beetroot‐related food/supplements depends on raw beetroot, processed products, and processing methods, which might slightly alter the efficacy of beetroot variants.

In summary, BRJ ingestion improves vascular endothelial dysfunction associated with aging, at least in part, by enhancing NO bioavailability and reducing oxidative stress; however, it does not suppress medial thickening. Dietary intake of beetroot could be a simple strategy to prevent age‐related vascular dysfunction.

## AUTHOR CONTRIBUTIONS

Masashi Tawa conceived and designed the experiments, performed the experiments, and analyzed the data. Masashi Tawa, Keisuke Nakagawa, and Mamoru Ohkita wrote the paper.

## FUNDING INFORMATION

This work was financially supported by the Grants‐in‐Aid for Scientific Research Program from the Japan Society for the Promotion of Science (grant number 22 K15299).

## CONFLICT OF INTEREST STATEMENT

The authors confirm that there is no conflict of interest related to the manuscript.

## Supporting information


Table S1.

## Data Availability

The data that support the findings of this study are available from the corresponding author upon reasonable request.
