# Supplementary material for: Effects of beetroot juice supplementation on vascular functional and structural changes in aged mice
Source: Physiol Rep. 2023 Jun 20;11(12):e15755. doi: 10.14814/phy2.15755 (PMC10281958; doi:10.14814/phy2.15755)
Supplement: Supplementary file 1 — Table S1. [file PHY2-11-e15755-s001.docx]

**Table S1.** Daily nitrate intake for each week in BRJ-supplemented aged mice. Data are the mean ± SEM values of 10 experiments. Abbreviations: BRJ, beetroot juice.

|  | 1st week | 2nd week | 3rd week | 4th week |
| --- | --- | --- | --- | --- |
| Nitrate intake  (μmol/kg/day) | 573.9 ± 23.3 | 531.9 ± 9.7 | 498.6 ± 7.4 | 478.7 ± 7.4 |
